# Dr. Thorne Thorne on Diphtheria

**Published:** 1891-10-17

**Authors:** 


### The Hospital

A Weekly Journal of

Science, Medicine, Nursing, and Philanthropy.

*For Hospitals, Asylums, Infirmaries, Dispensaries, Medical Practitioners, Students, Nurses, and the Charitable Public.*

Vol. XI.—No. 264.] [Oct. 17, 1891.

### Dr. Thorne Thorne on Diphtheria.

Medical men, not unnaturally, concern themselves principally with the treatment of disease. Of late years the better educated and more philosophical medical man has set himself to discover the nature of different maladies. Still more recently the physician with an element of statesmanship in him has made it his business to trace out the remote as well as the immediate causes of dangerous morbid processes, and so far as is possible to remove those causes or to check their activity. Dr. Thorne Thorne, who has just published his "Milroy" lectures on Diphtheria, has furnished the world with a most important contribution to its practically useful knowledge. All classes of readers would do well to make themselves acquainted at least with Dr. Thorne's reasoned conclusions. His book, although primarily addressed in the form of lectures to the fellows and members of the Royal College of Physicians, is by no means a book which concerns them entirely or mainly. On the contrary, it is eminently a book for all fathers and mothers, school teachers and school managers, statesmen, policemen, and sanitary inspectors, and indeed for all sorts and conditions of men and women who have any sort of responsibility for children and young people in either a public or a private capacity.

Diphtheria is a hateful, insidious, and deadly enemy to man and domestic animals. Familiarity with it breeds apathy in some medical minds; and limited and distorted knowledge gives rise to hopeless terrors in the minds of the public, and of parents who have delicate families. The apathy of the average doctor is discreditable to him; and the little distorted knowledge which the public possess is the cause of many avoidable calamities, and is much to be deplored. It is hardly likely that we shall succeed in persuading many of our non-medical readers to buy, and intelligently study Dr. Thorne Thorne's book; but they may be induced to consider two or three points of great practical importance, if those points are put before them in a brief and simple form.

To begin with, let us ask ourselves what the houses in which we live may have to do with causing diphtheria? It is probable that they sometimes have everything to do with it. A house built in a low-lying situation, where the ground underneath and about it is constantly soaked with water from the falling rain, provides the very conditions which the bacillus of diphtheria seems to choose by preference for its active development. Dr. Thorne Thorne is convinced that true diphtheria is a bacillary disease. Now let any intelligent man betake himself to a house, which has been unoccupied for some time, which is situated on low-lying ground, or on a cold northerly slope, surrounded perhaps by thick belts of trees. Let him examine the inner and outer walls, more especially those that are clothed with ivy, or have for some reason become sodden and damp. He will find almost all those places covered with green moulds or other fungiform growths in the highest state of activity. If human beings had lived in the house their fires and brooms might have kept the walls clear of moulds and fungi, but the damp, cold atmosphere within and around would still have been present, and the sore and relaxed throats which are so prevalent in cold and damp situations would have offered precisely the same invitation to the diphtheria bacillus to settle and grow upon them as the damp and unswept walls of the empty house offered to the moulds and fungi. This is no mere theory. Dr. Thorne Thorne, who has accumulated his facts with rare industry, and studied them with admirable intelligence, is convinced that low-lying damp neighbourhoods and cold and wet exposures are among the most favourable of all conditions for outbreaks of diphtheria.

The sceptical reader, who still pins his faith to bad drains and insanitary conditions as the main cause of diphtheria, should take seriously into consideration one or two very striking facts. For example, twenty years ago diphtheria was three times as prevalent, and three times as fatal in country districts as in large towns. But nobody would assert that the sanitation, or at least the air, of towns was purer than that of country places. The opposite indeed was, and still is, the truth. So far as mere purity is concerned country air has always had the advantage over that of towns. But villages and outlying farms and cottages have always been much more exposed to cold and damp than the streets of towns. It is a reasonable conclusion, therefore, that since villages twenty years ago suffered much more than towns, cold and damp, and not merely impure air, have had much to do with the origin and spread of diphtheria in those places. The obvious inference is that we should as far as possible live in houses that stand on high and dry situations, that have sunny exposures, and that are so built as to admit of every apartment both great and small being flooded with sunshine at some time or other of the day.

But although diphtheria may thus apparently arise *de novo* from unfavourable atmospheric conditions, Dr. Thorne Thorne is emphatically of opinion that there are at least two other sources which markedly tend to promote its spread. Those two sources are uncooked milk, and public elementary schools. There appears to be no doubt that the domestic cow, like the cat and the tenants of the fowl-house, is more or less frequently attacked by diphtheria. When it is considered that cows sleep out-of-doors all night, not only during the spring and summer, but also in the cold month of October, and when it is also remembered that cow pastures are often by preference selected in low-lying and damp ground because of the abundance of grass which grows in such situations, and the plentiful supply of water that is usually to be found in them, it may well be that diphtheria is a much more common disease among cattle than any of us have hitherto supposed. This is a subject which would well repay investigation.

There seems to be no doubt that the milk of cows suffering from diphtheria will communicate the disease to those who drink it unless it be thoroughly cooked. If this be so, and Dr. Thorne Thorne offers evidence of a very convincing kind on the point, what must be thought of those parents who, to save themselves or their servants a little trouble, will not be at the pains to insist that all the milk of the family shall be boiled before it is used? Many people are under the impression that milk boiled is milk spoiled. But if the milk be boiled immediately after it is received from the milkman, and then allowed to cool before it is used, its flavour is in no way interfered with. Milk ought no more to be taken raw than beef or pork. It may be that the raw milk which was universally used in the country twenty years ago, and in double or treble the quantities taken by town-folk, had a great deal to do with the increased prevalence of diphtheria in country districts.

Public elementary schools are believed by Dr. Thorne Thorne to have been responsible for some very severe epidemics of diphtheria during the past twenty years. In that period a very remarkable coincidence of circumstances has been observed. In 1870 Mr. Forster's Elementary Education Act was passed, and its effect was to sweep into the public elementary schools all the children of the poorest, worst fed, worst housed, and most unhealthy classes of the population. Within the same period, diphtheria has so rapidly increased in large towns that now the number of cases per thousand is almost on a level with that of country places. This is not due, as might be supposed, to any increasing general unhealthiness in towns. For it is well known that urban sanitation is remarkably improved within the last twenty years, and the proof of its improvement is at hand in the undoubted fact that typhoid fever and other zymotic diseases have rapidly diminished both in their extent and their severity during that time. But Dr. Thorne Thorne does not rest his conclusion on a mere coincidence of this kind. He has abundant evidence to show that sore throats and other conditions which conduce to the development of diphtheria prevail very extensively, both among the teachers and the pupils in those public elementary schools that are situated in the poorest neighbourhoods. Not only so, but some of the severest epidemics of recent times have had their focus of extension traced directly to certain schools.

Here, then, we have a body of undoubted facts to deal with—facts of the plainest and most easily understood kind. Everybody can at least try to find a home at a good elevation, on dry ground, and with a sunny exposure. And everybody can do more than try to boil milk before using it—they can boil it, and that without any trouble at all. With regard to public elementary schools, it is obvious that a thorough inquiry must be made into their condition and affairs by Government authority, and efficient steps must be taken, at least to prevent them from becoming centres of dissemination for such a dangerous disease as diphtheria. There is no doubt whatever that Dr. Thorne Thorne has put his finger upon some of the weak points in the armour of our civilisation. It is for us as a practical people to make use of the knowledge which his experience and opportunities at the Local Government Board have enabled him to gather, and which he has so clearly and intelligently set forth.

